# Transformation of siderite to goethite by humic acid in the natural environment

**DOI:** 10.1038/s42004-020-0284-3

**Published:** 2020-03-25

**Authors:** Bobo Xing, Nigel Graham, Wenzheng Yu

**Affiliations:** 1 Key Laboratory of Drinking Water Science and Technology, Research Center for Eco-Environmental Sciences, Chinese Academy of Sciences, 10086 Beijing, China; 2 Department of Civil and Environmental Engineering, Imperial College London, South Kensington Campus, London, SW7 2AZ UK

**Keywords:** Geochemistry, Nanoparticles

## Abstract

Humic acid (HA) is particularly important in iron-bearing mineral transformations and erosion at the water-mineral boundary zone of the Earth. In this study, three stages of the possible pathway by which HA causes mineral transformation from siderite to goethite are identified. Firstly, a Fe(II)-HA complex is formed by chelation, which accelerates the dissolution and oxidation of Fe(II) from the surface of siderite. As the Fe(II)-HA complex retains Fe atoms in close proximity of each other, ferrihydrite is formed by the agglomeration and crystallization. Finally, the ferrihydrite structurally rearranges upon attachment to the surface of goethite crystals and merges with its structure. The influence of low concentrations of HA (0–2 mg/L) on phosphate adsorption is found to be beneficial by the inducing of new mineral phases. We believe that these results provide a greater understanding of the impact of HA in the biogeochemical cycle of phosphate, mineral transformation.

## Abstract (web summary)

The biochemical phosphate cycle is dependent on interfacial reactions between humic acid and iron-bearing minerals, but the role of humic acid remains unclear. Here the authors study the reaction of siderite with humic acid and its influence on phosphate adsorption.

## Introduction

Iron is the fourth most abundant element, which can form complexes with elements such as C, N, O, and S, making it an essential element for nearly all living organisms^1–7^. The environmental iron-bearing mineral phases (such as the iron oxides) are aggregates of nano-particles whose performance of adsorption and release of nutrients and metals are highly dependent on the particles size, crystallinity, and transformation product^8,9^. Hence, it is important to understand and reveal the transformation and crystallization mechanisms of such minerals in the natural environment.

Currently, Ostwald ripening^10^ and Oriented attachment^11–14^ are widely accepted as possible crystallization mechanisms^15–19^. However, there are many interplaying factors that remain unknown, particularly the interfacial forces and nucleation mechanism^11,12^. The magnitude of the free-energy barrier of nucleation is a significant factor in determining the properties and the number of particles produced^20–22^. At low concentrations, temperatures, and pressures, the free-energy barrier is relatively large^17–19,23^, so the processes of nucleation and growth should be rare in the natural environment in those predicted by classical models^19,24^. However, mineral transformation happens in nature all the time, but the crystallization path in the natural environment is not well established and needs further investigation.

The previous research demonstrates that the precursor phase is a ubiquitous feature of crystallizing systems^15,25^. Consequently, the pathways of crystals nucleation and growth often relate to the precursor particles. In biomineralization, organic matter (OM) enables the efficient transport of multi-ion complexes with low concentration to the crystallization site by precursor particles^16^. Several mechanisms of crystal nucleation and growth induction by OM have been proposed, including direct attachment and nucleation in a pre-aligned OM^26^, such as collagen^27^, or alignment through physical interactions^28^. OM also has been shown to modulate the kinetics of multi-ion complexes nucleation and growth. Macromolecules (proteins, polyacrylic acid, and humic acid) can dramatically stabilize amorphous precursors^27,29^, increase induction times^30^, induce the formation of dense liquid phases^31^, and modify crystal shape and size^32–36^.

Previous research mainly focused on the synthesis of nanomaterials, so the crystals and OM selected in their experiment is not abundant in natural water or soil^12,37^. To reveal the transformation and crystallization mechanisms of minerals in natural environment, it is necessary to select common substances in natural water or soil as research objects^38^. Due to the redox reaction between Fe(ІІ) and Fe(ІІІ) represents one of the most important factors affecting the biogeochemical cycle of nutrients and metals as well as environmental remediation, it is particularly important to select the divalent iron-bearing mineral as research object. Siderite (FeCO_3_) is particularly abundant in China with reserves up to 2 billion tons^18^, which can be used as a representative of divalent iron-bearing mineral in soil to investigate. Meanwhile, goethite (α-FeOOH) is one of the most abundant of the natural Fe(ІІІ) iron-bearing mineral in soils, and it has a high percentage of the total surface area of the soil (more than 50%) due to its nanoparticle size and it as a coating on other soil minerals^39^. The knowledge of the transformation from siderite to goethite, especially the OM influence on transformation, is critical for the understanding the mechanism of mineral transformation in the environment. Meanwhile, humic acid (HA) is a representative natural OM and a most frequently found organic compound in soil, possessing abundant hydroxyl and carboxylic groups that facilitate its adsorption onto minerals and its role in mineral transformation^40^. Although geological materials provided early examples of crystal nucleation and growth induction by HA^40,41^, the role of HA in this process remains uncertain and requiring further investigation.

Here, we investigate the size, morphology, and crystallinity of synthesized siderite and its products after reacting with HA (different concentration). To explore the different transformation paths caused by different precursor particles, the oxidation of siderite by H_2_O_2_ and dissolved oxygen (DO), and the influence of HA on the transport of phosphate, are considered and compared. The experimental results obtained help to explain the possible pathway of siderite mineral transformation by humic acid and its influence on the adsorption of phosphate. Thus, the results provide new information about the role of humic acid in the biogeochemical cycle of phosphorus, siderite mineral transformation, and its significance in environmental remediation.

## Results and discussion

### Morphology and crystallinity of synthesized siderite

**Fig. 1:**
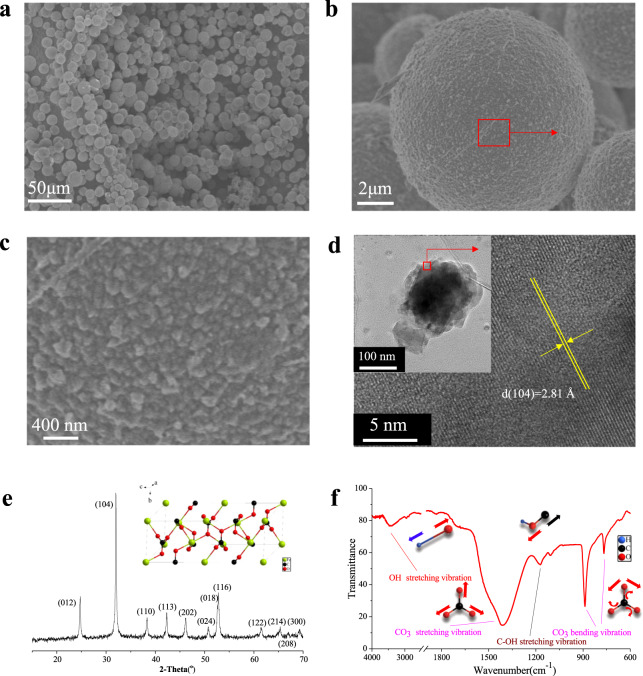
Characterization of synthesized siderite. **a** Representative SEM images of the synthesized siderite obtained through hydrothermal reaction at 130 °C for 4 h, and the more highly magnified SEM images of an individual microsphere (**b**) and its surface morphology (**c**); **d** representative TEM (inset of **d**) and HRTEM (**d**) of the nanoparticle on the surface of synthesized siderite; **e** the XRD pattern of the synthesized siderite, where the inset is the schematic diagram of the siderite unit cell structure, which was drawn by use of Diamond 3.2 software (Crystal Impact GbR, Germany); **f** the FTIR spectra of synthesized siderite.

The size and morphology of the synthesized siderite were observed (Fig. 1a–c). A large number of siderite spheres (Fig. 1a) were obtained through the hydrothermal reaction (130 °C for 4 h, diameters range from 5 to 20 μm). A single siderite sphere is shown in Fig. 1b with a large quantity of submicrometre-sized polyhedral subunits (Fig. 1c) on its surface. This suggests that the siderite spheres are formed by a process of subunit aggregation. There are many studies concerning models for the aggregation of microspheres^42–44^. In this study, the sizes of polyhedral subunits substances ranged from 100 to 200 nm (inset of Fig. 1d), which is consistent with the size observed by SEM (Fig. 1c). To confirm the composition of the sphere, the lattice fringes of the polyhedral subunits were investigated by HRTEM (Fig. 1d), and its lattice fringes were calculated as 2.81 Å, which coincided with the (104) interplanar distance of siderite (inset of Fig. 1e). Therefore, the composition of the sphere substance (Fig. 1a) was siderite.

Meanwhile, the crystallinity of synthesized siderite was investigated and the XRD patterns are shown in Fig. 1e. The main XRD diffraction peaks were located at 2*θ* = 24.5°, 32°, 38°, 42°, 46°, 51°, 52.5°, 61.5°, and these were ascribed to siderite (ICDD (29-0696)). All diffraction peaks were sharp and strong, and no additional peak was detected, indicating that the obtained siderite was well crystallized. This result is consistent with HRTEM (Fig. 1d) analysis. The vibration mode of the functional groups of siderite is shown in Fig. 1f. The detailed evidence of siderite growth process is shown in Supplementary Methods and Supplementary Fig. 1.

### Mineral transformation from siderite to goethite induced by humic acid

**Fig. 2:**
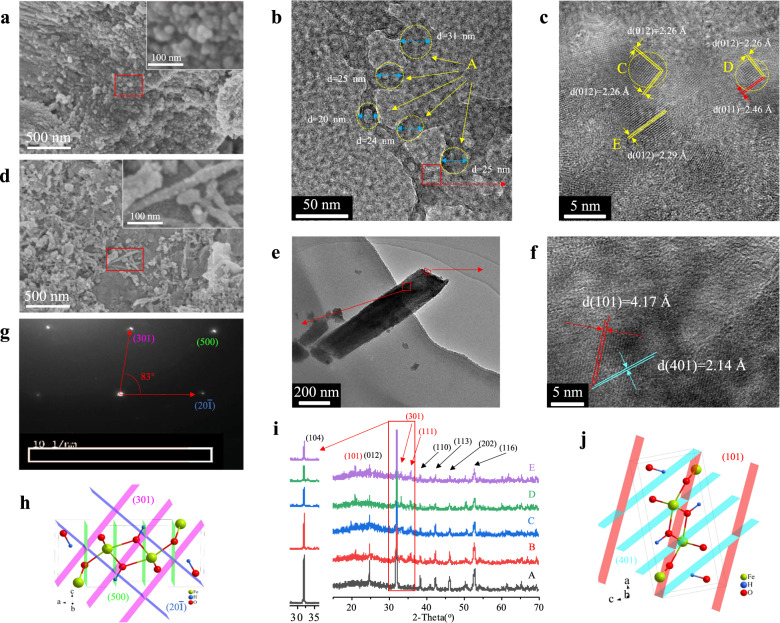
Characterization of the precipitates. Representative SEM (**a** and **d**) and TEM (**b** and **e**), HRTEM (**c** and **f**), and SAED (**g**) images of the precipitates of siderite after reacting with HA (2 mg/L); the XRD (**i**) pattern of the synthesized siderite after reacting with 0 mg/L (**i**-A), 0.5 mg/L (**i**-B), 1 mg/L (**i**-C), 2 mg/L (**i**-D) and 10 mg/L HA (**i**-E); schematic diagrams of goethite (**h** and **j**) unit cell structures, which were drawn by the Diamond 3.2 software (Crystal Impact GbR, Germany). Samples were freeze-dried for 12 h after 7 days of aging. The concentration of dissolved oxygen (DO) were 4 mg/L.

To provide direct evidence of humic acid-induced mineral transformation from siderite to goethite, the product of siderite after reacting with HA (2 mg/L, DO: 4 mg/L) was carefully investigated by SEM, TEM, HRTEM, XRD and selected area electron diffraction (SAED). Representative images of samples are shown in Fig. 2a–j.

A large number of particulate substances were observed in the precipitate of siderite after reacting with HA (Fig. 2a–d). Sphere-shaped (inset of Fig. 2a) and rod-shaped (inset of Fig. 2d) substances were observed in the more highly magnified SEM images. The morphologies of the precipitate are different from the submicrometre-sized polyhedral subunits (Fig. 1c) on the surface of synthesized siderite, which implied that new mineral phases had been formed in the reaction between siderite and HA.

To further confirm the composition of sphere-shaped (inset of Fig. 2a) and rod-shaped (inset of Fig. 2d) substances. The precipitates of siderite after reacting with HA were observed by TEM (Fig. 2b–e) and HRTEM (Fig. 2c–f). The average sizes of sphere-shaped substances ranged from 20 to 30 nm (region A in Fig. 2b and Supplementary Fig. 2a, b), which is consistent with the size observed by SEM (inset of Fig. 2a). The lattice fringes of the sphere-shaped (Fig. 2b) was observed by high-resolution TEM (Fig. 2c). Both of the two interplanar spacings in region C (Fig. 2c) were 2.26 Å, the calculated (012) lattice planes and the angle between them, indicated that the sphere-shaped substance was a twin crystal of ferrihydrite, which is similar to the region D (Fig. 2c). Due to small misalignments during attachment, the twin crystal is formed, which can also be eliminated through the rearrangement or recrystallization of primary particles after their aggregation (such as region E in Fig. 2c)^15,16,20,25,45–47^.

Meanwhile, the rod-shapes is clearly visible by the highly magnified TEM images shown in Fig. 2e and Supplementary Fig. 2b, c. The HRTEM image of the vertex outlined in Fig. 2e is displayed in Fig. 2f. The resolved interplanar spacings were 4.17 Å and 2.14 Å, which corresponded to the (101) and (401) lattice planes of goethite, respectively. The calculated (101) and (401) lattice planes and the angle between them coincide with the goethite unit cell structure (Fig. 2j). The indexed regular SAED spots demonstrated that the rod-shaped substance was a single crystal of goethite (Fig. 2g). The calculated (301), (500), and (20$\bar 1$) lattice planes also coincided with the goethite unit cell structure (Fig. 2h). The above analysis confirmed that the rod-shaped substance was goethite, and its growth orientation was nearly perpendicular to the b-axis direction. Therefore, it was concluded that HA can induce the mineral transformation from siderite to goethite.

Although these results provide direct evidence of the formation of goethite (more evidence of goethite and siderite are shown in the Supplementary Fig. 2), the common phenomenon of mineral transformation required further analysis. Therefore, the precipitates of synthesized siderite after reacting with different concentration of HA (0, 0.5, 1, 2, and 10 mg/L; DO: 4 mg/L) were investigated by XRD and the patterns are shown in Fig. 2i. The main XRD diffraction peaks located at 2*θ* = 24.5°, 32°, 38°, 42°, 46°, 51°, 52.5°, 61.5° were ascribed to siderite (ICDD (29-0696)). Although the diffraction intensity of siderite was different after reacting with different concentration of HA, the main component of synthesized siderite after reacting with HA was still siderite. However, the rest diffraction peaks located at 2*θ* = 17.8°, 21.2°, 33.2°, 34.6°, and 36.6° were evident in Fig. 2i (B–E), which corresponded to goethite (ICDD (29-0713), [space group *Pbnm* (62)]) with lattice constants *a* = 9.91 Å, *b* = 3.01 Å, and *c* = 4.58 Å. All diffraction peaks of goethite were weak and gentle, indicating that the produced goethite had a low degree of crystallization. However, the XRD (spectrum D in Fig. 2i) diffraction peaks of the precipitate of HA (2 mg/L) were different from the others. The diffraction peak located at 2*θ* = 33.2° was significantly higher than the peak located at 36.6°, and the relative strength was consistent with the characteristic diffraction peaks of goethite (ICDD (29-0713)), which revealed that the induction degrees from siderite to goethite by different concentration of HA was different, and HA (2 mg/L) had the highest inductive ability.

Combining the results of the XRD, SEM, and TEM analyses, the rod-shaped substances (inset of Fig. 2d) were identified as goethite. Considering that no additional peak was detected in the XRD pattern (Fig. 2i), it was concluded that the sphere-shaped substances (inset of Fig. 2a) were X-ray poorly crystalline ferrihydrite.

In conclusion, HA can induce mineral transformation from siderite to goethite in natural environment, and as a precursor, ferrihydrite exists in the mineral transformation process. The role of HA in this reaction needs to be further investigated.

### Crystal growth and polymerization process

In-situ observations of crystal nucleation and growth from solution at an atomic-scale lattice resolution are rare, and it is generally investigated by liquid phase scanning probe^48^ and transmission electron microscopy (TEM)^13,49,50^. Consequently, there are very few systems which can unequivocally demonstrate the process of mineral transformation. Nonetheless, TEM has been frequently accepted as a characterization method of mineral transformation.

**Fig. 3:**
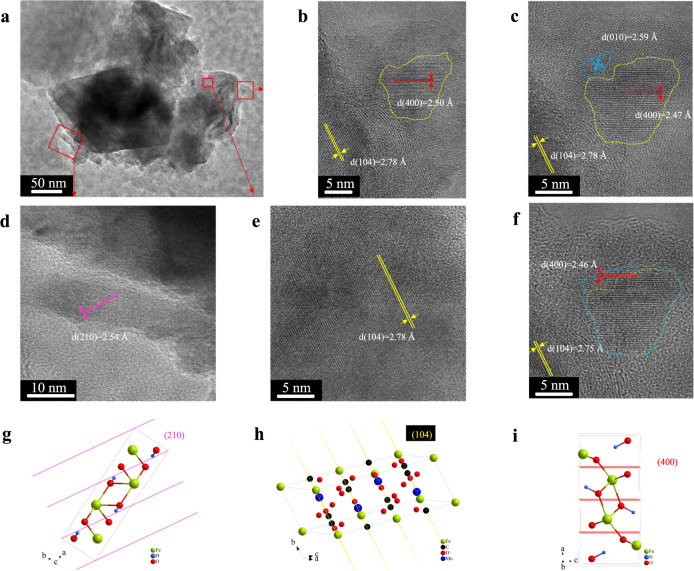
Characterization of mesocrystal. Representative TEM (**a**) and HRTEM (**b**–**f**) images of the precipitate of siderite after reacting with HA; schematic diagrams of goethite (**g** and **i**) and siderite (**h**) unit cell structures, which were drawn by Diamond 3.2 software (Crystal Impact GbR, Germany). The lattice fringes of the yellow region in Fig. 3b was observed after 1 min (Fig. 3c) and 2 min (Fig. 3f), respectively. Samples were freeze-dried for 12 h after 1 days of aging.

To explore the growth and polymerization process of goethite, the lattice fringes of the precipitates (after aging for one day) of siderite-HA were investigated by HRTEM (Fig. 3a–i). One large particulate substance was observed (Fig. 3a), and its lattice fringes were calculated as 2.78 Å (Fig. 3e), which coincide with the siderite unit cell structure (Fig. 3h). Therefore, the main composition of the particulate substance (Fig. 3a) was siderite. The edge of siderite was observed by high-resolution TEM (Fig. 3b), and the lattice fringes of goethite (*d*(400) = 2.50 Å), which coincide with the goethite unit cell structure (Fig. 3i)) were observed in the yellow region. After 1 min, lattice fringes of ferrihydrite (blue region, *d*(010) = 2.59 Å) were observed at the upper left of the yellow region (Fig. 3c). After 2 min, the ferrihydrite and goethite merged in the blue area (Fig. 3f), which suggested that the nano-particles structurally rearranged upon attachment to the surface of goethite crystals to merge with the goethite crystal structure. The lower-left corner of siderite (Fig. 3a) was observed by high-resolution TEM (Fig. 3d), and the rod-shaped goethite (*d*(210)=2.54 Å), which coincided with the goethite unit cell structure (Fig. 3g)) was just beginning to grow.

In conclusion, the growth and polymerization process of goethite follow the Oriented Attachment (OA) mechanism, particles coalesce or aggregate to form larger particles with pre-aligned crystallographic orientations^11–14^. Whereas previous research clearly demonstrates crystallization by OA, where many interplaying factors are still unknown^11,12^. However, previous research put forward the supposition that the mesocrystal is a necessary precursor to the single-crystal attachment, but is also a fundamental step for crystal growth^28,51^. Therefore, in this study, ferrihydrite as a precursor (mesocrystal) may be one of the important reasons for mineral transformation.

### The important role of mesocrystal (ferrihydrite) in mineral transformation from siderite to goethite

**Fig. 4:**
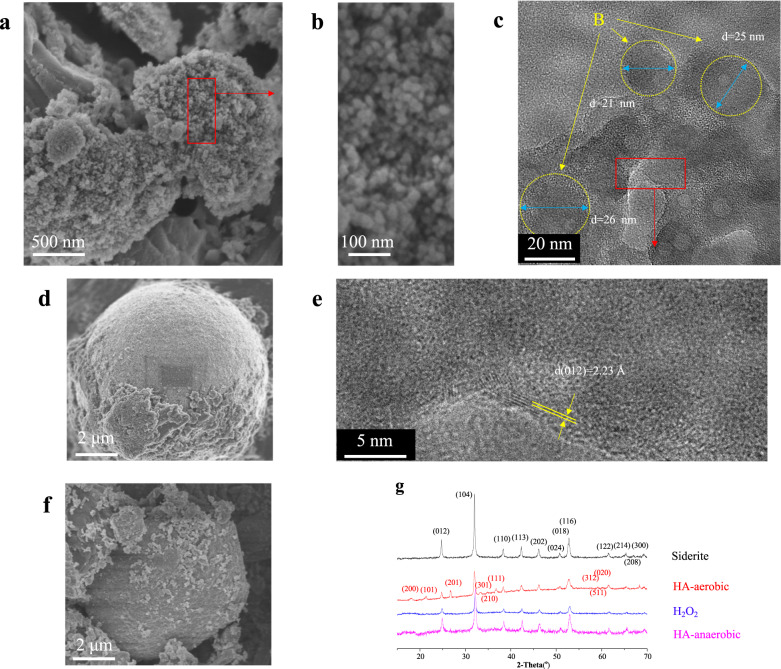
Characterization of control products. Representative SEM images of the synthesized siderite after reacting with H_2_O_2_ (**a**, **b**), H_2_O-DO (**d**) and HA-anaerobic (**f**); representative TEM (**c**) and HRTEM (**e**) images of the synthesized siderite after reacting with H_2_O_2_; (**g**) The XRD pattern of the synthesized siderite and the precipitates. Samples were freeze-dried for 12 h after 7 days of aging.

To reveal the role of ferrihydrite and the mechanism of HA-induced mineral transformation, the transformation results (processes, intermediates, and the final product) of siderite by HA (aerobic and anaerobic, respectively) and oxidant (H_2_O_2_) were compared by SEM and XRD analysis (Fig. 4a–g). The two simulated solutions of HA are separately defined as HA-aerobic and HA-anaerobic. The HA discussed in the previous section is HA-aerobic.

The precipitates of synthesized siderite after the reaction were investigated by XRD and the patterns are shown in Fig. 4g. The main XRD diffraction peaks located at 2*θ* = 24.5°, 32°, 38°, 42°, 46°, 51°, 52.5°, and 61.5° were ascribed to siderite (ICDD (29-0696)). This indicated that the main component of synthesized siderite after reacting with HA-aerobic, HA-anaerobic, and H_2_O_2_ was still siderite. However, the XRD diffraction peaks of the precipitate of HA-aerobic were different from the others. The rest diffraction peaks located at 2*θ* = 17.8°, 21.2°, 33.2°, 34.6°, and 36.6° were evident in Fig. 4g (HA-aerobic), the previous section revealed the product of siderite after reacting with HA-aerobic (Fig. 2a–d) was sphere-shaped ferrihydrite and rod-shaped goethite. The XRD results reveal that only HA-aerobic can induce siderite to goethite, so whether their weakly crystalline intermediates are different?

**Fig. 5:**
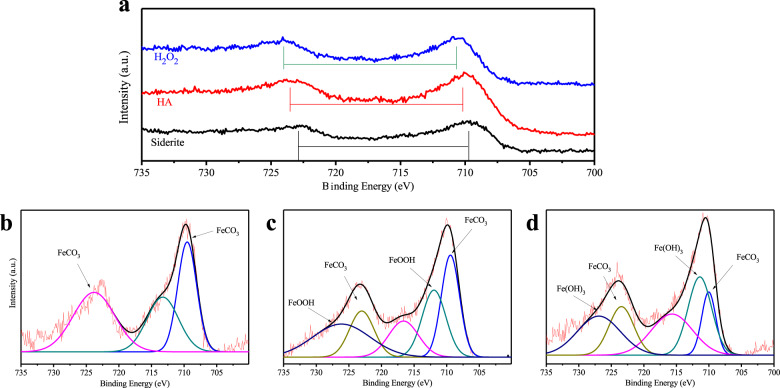
The XPS spectra of Fe 2p. The survey and high-resolution scans of XPS spectra of siderite after reacting with HA and H_2_O_2_. **a** Total survey scans of Fe 2p; **b** Fe 2p peaks of siderite; **c** Fe 2p peaks of siderite-HA; **d** Fe 2p peaks of siderite-H_2_O_2_. Samples were freeze-dried for 12 h after 7 days of aging.

Furthermore, a mass of sphere-shaped substances (Fig. 4a, b) were observed in the precipitate of siderite after reacting with H_2_O_2_, as a comparison the size of these was similar to the sphere-shaped substances of siderite-HA (inset of Fig. 2a), suggesting that new mineral phases also formed in the reaction between siderite and H_2_O_2_. The lattice fringes of the sphere-shaped substances of siderite-H_2_O_2_ (Fig. 4b) were also observed by high-resolution TEM (Fig. 4b–e). In contrast, there were only a few lattice fringes that could be observed at the edge of the sphere-shaped substances. Meanwhile, the total survey scans of XPS spectra of Fe 2p (Fig. 5a) and O 1s (Supplementary Fig. 3) demonstrate the effect of HA and H_2_O_2_ on the siderite. The Fe (2p3/2)/Fe (2p1/2) spectra (Fig. 5a) of siderite and siderite-HA can be seen at 709.1/722.5 eV and 710.2/723.3 eV, respectively, which indicated that the precipitates of the siderite after reacting with HA were different with siderite. To reveal the reaction mechanisms, the detailed Fe 2p analyses of siderite (Fig. 5b) after reaction with HA are shown in Fig. 5c. The Fe 2p peaks that are assigned to FeCO_3_ display broad Fe 2p3/2 and Fe 2p1/2 lines located at 709.8 ± 0.2 and 724.5 ± 0.2 eV, respectively (Fig. 5b). The peak at 711.5 ± 0.2 eV of siderite after reacting with HA is assigned to the formation of goethite (Fig. 5c)^52^. The peak of binding energy at 712.3 ± 0.2 eV (Fig. 5d) of siderite, after reaction with H_2_O_2_, is higher than that of siderite after reaction with HA (711.8 ± 0.2 eV), which result from the formation of Fe(OH)_3_^52^. Therefore, it appeared that H_2_O_2_ could only oxidize siderite into amorphous Fe (ІІІ) oxyhydroxides (Fe(OH)_3_), indicating that it was difficult to promote the further crystallization of Fe(OH)_3_. However, the sphere-shape of the siderite-HA consisted of ferrihydrite nanoparticles, which were present as mesocrystals in the process of mineral transformation.

However, particulate substances (the remains of the spheres, Fig. 4f) with a larger size ranging from 5 to 10 μm (approximately half the size of the synthetic siderite) were observed in the sediment of siderite after reacting with HA-anaerobic; this suggested that the large particulate substances were the residue of siderite, indicating that the extent of erosion caused by HA (without DO) on siderite was minor. A comparative experiment with only dissolved oxygen (DO), and without HA, was also investigated, and the result showed that the morphology and crystallinity of the precipitates were almost unchanged (No new crystals were formed, Fig. 4d). From this observation, it was inferred that chelation can take place between humic acid and the released ferrous ions to form a Fe(II)-HA complex, which could accelerate the dissolution of siderite.

In conclusion, although humic acid cannot directly oxidize siderite, it can chelate with the released ferrous ions to form a Fe(II)-HA complex, accelerating the oxidation of Fe(II) by DO in water and forming the mesocrystals (ferrihydrite), and further, induce the formation of goethite.

### The migration path of HA during mineral transformation

Despite the successes of classical nucleation and growth models^53,54^, there are several phenomena associated with crystal formation that cannot be explained satisfactorily or predicted either quantitatively or qualitatively.

**Fig. 6:**
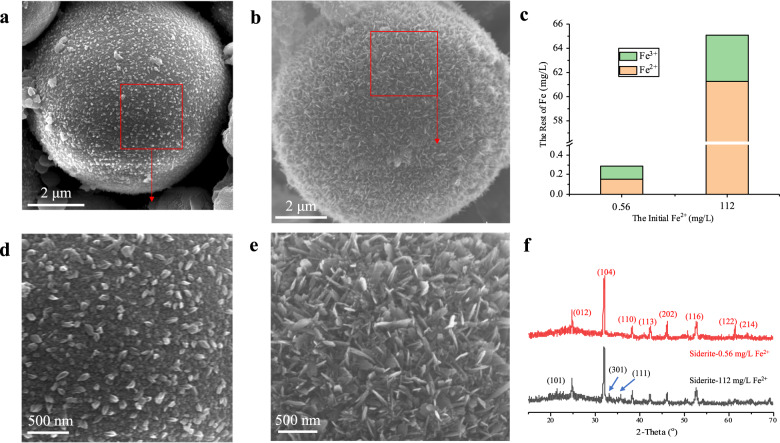
Control products of Fe^2+^. Representative SEM images of the synthesized siderite after reacting with 0.56 mg/L (**a**, **d**) and 112 mg/L (**b**, **e**) Fe^2+^; **c** the rest concentration of Fe after reaction; and **f** the XRD pattern of the synthesized siderite after reacting with different concentration Fe^2+^. Samples were freeze-dried for 12 h after 7 days of aging.

Shu et al.^55^ revealed that Fe^2+^ (2 mM) in the solution could induced the transformation of ferrihydrite. To reveal the mineral transformation mechanisms in the presence of HA, and the possible effects of Fe^2+^ need to be taken into account. Therefore, the concentration of Fe^2+^ and total Fe of the filtrates (siderite after reacting with HA at different concentrations) were measured (Supplementary Fig. 4). The results revealed that the concentration of Fe^2+^ and total Fe were ~0.15 mg/L and 0.27 mg/L, respectively. Therefore, the transformation results of siderite (without HA) by two different concentration Fe^2+^ (0.1 mM (0.56 mg/L, approximately two times than this study) and 2 mM (112 mg/L, the same as Shu et al.^55^) were compared by SEM and XRD analysis (Fig. 6a–f).

A large number of particulate (Fig. 6a–d) and plate-shapes (Fig. 6b–e) substances were observed on the surface of siderite after reacting with 0.1 mM and 2 mM Fe^2+^, respectively. The morphologies of the precipitate are different from the submicrometre-sized polyhedral subunits (Fig. 1c) on the surface of synthesized siderite, which implied that new mineral phases had been formed. The XRD results (Fig. 6f) revealed that only 2 mM Fe^2+^ could induce the transformation from siderite to goethite. Meanwhile, the concentration of Fe^2+^ had halved after the reaction (Fig. 6c), which indicated the goethite formed by the added Fe^2+^, consistent with the results by Su et al.^55^. However, the low concentration of Fe^2+^ (it shall not be higher than 0.1 mM) could not induce the transformation from siderite to goethite. Therefore, the possible effects of Fe^2+^ do not need to be taken into account in this study.

Combined with previous studies, we found that the Fe concentrations, temperature, and pressure requited for the nucleation of ferrihydrite in this study are well below those predicted by classical models^19,24^. To reveal the role of HA in this transformation, the filtrates (siderite after reacting with HA at different concentrations) were investigated by three-dimensional EEM fluorescence spectroscopy (Supplementary Fig. 5). In this study, the peak of the original HA appeared in the region of 200–300/350–550 nm (Ex/Em). For different concentrations of HA (0.5, 2, and 10 mg/L), the location of fluorescence peaks did not alter, but their intensity gradually decreased with the reaction time (Supplementary Fig. 5). The results implied that Fe(II) complexed primarily with the carboxyl functional groups of HA, which would lead to the formation of aggregates as contact time increased.

**Fig. 7:**
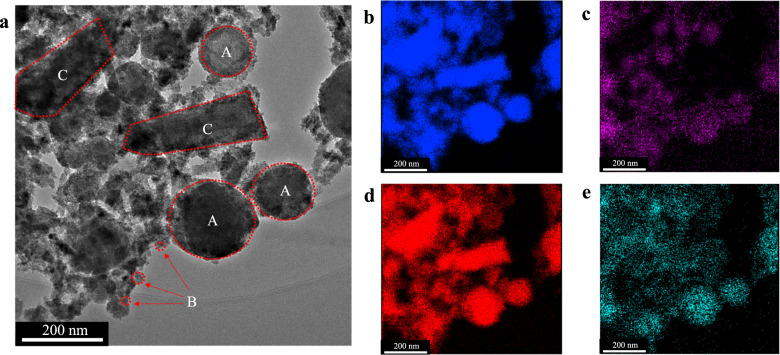
Element mapping of precipitate. Element mapping of siderite after reacting with HA and phosphate (**a** TEM; **b** Fe; **c** C; **d** O; **e** P). The initial concentration of P was 5 mg/L. Samples were freeze-dried for 12 h after 7 days of aging.

Furthermore, the element mapping of siderite-HA was investigated (Fig. 7a–e). Some substances of spherical, rod-like, and clusters of particles were evident in Fig. 7a. In order to distinguish the different structures in Fig. 7a, three regions from A to C were highlighted, where the crystals in the regions A and C are compact in the arrangement of elements Fe (Fig. 7b) and O (Fig. 7d). Based on the previous sections analyses, the substances of spherical (region A) and rod-like (region C) shapes are identified as siderite (FeCO_3_) and goethite (FeOOH), respectively. Simultaneously, the element C (Fig. 7c) was presented at regions A and B, which indicated that the elements Fe and C were the major mineralogical component for the particles in the region B. In addition, lattice fringes of ferrihydrite can be detected in the HRTEM images (Fig. 2c), demonstrating that the spherical substance in region B was ferrihydrite. It should be noted that the element C was rare in the region C (Fig. 7c), which implied that the newly formed goethite does not contain HA (element C). This suggested that the crystal growth will lead to the breaking of bonds between humic acid and Fe atoms and the subsequent expulsion of HA.

### Possible pathways of humic acid-induced mineral transformation from siderite to goethite

**Fig. 8:**
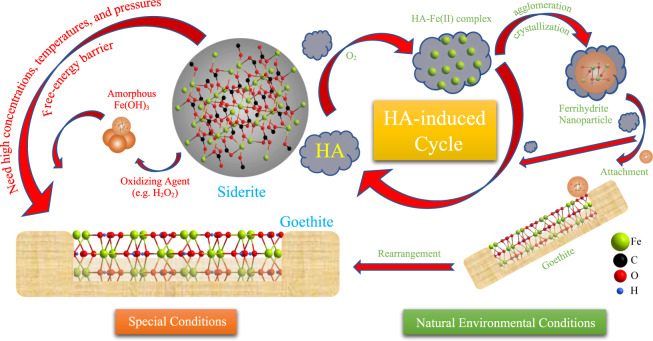
Path diagram of mineral transformation. Possible pathways of mineral transformation from siderite to goethite by humic acid.

The mineral transformation of synthesized siderite by humic acid was investigated using material microstructure characterization techniques, with the objective being to explain the possible pathway of humic acid-induced mineral transformation for siderite. It should be noted that similar mineral transformation studies have been done before, but the previous studies reveal that mineral transformation could only happen at high temperature (300 °C) and specific pH conditions (Fig. 8), which suggested that only the calcined siderite formed at high temperatures could be a good adsorbent^17^.

In this study, we have found that siderite can spontaneously transform to goethite in the presence of organic matter such as humic acid. The mineral transformation can happen without the harsh experimental conditions mentioned above. On the contrary, there was no rod-shaped substance (goethite) detectable in the precipitates of synthesized siderite after reacting with H_2_O_2_ (Fig. 4a, b), which demonstrated that the mineral transformation from amorphous Fe (ІІІ) oxyhydroxides (Fe(OH)_3_) to goethite (Fig. 4g) was extremely slow, or did not occur without the above-mentioned harsh experimental conditions. The major difference is that humic acid is able to chelate with the released ferrous ions to form a Fe(II)-HA complex, which accelerates the dissolution and oxidation of Fe(II) from the surface of siderite; and then owing to the Fe(II)-HA complex keeping Fe atoms within the effective distance of each other, the formation of ferrihydrite (region B in Fig. 7a) occurs by the agglomeration and crystallization of Fe atoms. In biomineralization, transient amorphous precursor particles (mesocrystals) enable the efficient transport of multi-ion complexes in low concentration to the crystallization site. Hence, with the effect of humic acid, the primary nanocrystals achieve crystallographic alignment despite spatial separation from each other.

In this study, it was concluded that the transformation from siderite to goethite in the presence of humic acid can be divided into three stages. Initially, humic acid chelates the released ferrous ions to form a Fe(II)-HA complex; and then the ferrihydrite was formed by the oxidation and crystallization of Fe(II); finally, the ferrihydrite, which is present as mesocrystals, structurally rearrange upon attachment to the surface of goethite crystals to merge with the goethite crystal structure. The breaking of bonds between humic acid and Fe atoms occurs during the crystal growth, followed by the expulsion of the HA, which then is available to chelate with fresh Fe(II). This possible pathway of humic acid-induced the mineral transformation from siderite to goethite in natural environment are shown in Fig. 8.

### The impact of mineral transformation for the biogeochemical cycle of phosphate

The mineral transformation has special significance for the biogeochemical cycle of nutrients and environmental restoration in water and soil systems. There is a competitive adsorption relationship between HA and phosphate, but in this study, HA can induce mineral transformation, and the phosphate adsorption may be promoted by the new minerals (ferrihydrite and goethite), so the adsorption of phosphate during the process of mineral transformation was investigated (Supplementary Fig. 6a–e).

In this study, both the effect of the HA concentration (Supplementary Fig. 6b) and reaction time (Supplementary Fig. 6a) on the adsorption of phosphate by synthesized siderite were considered. It was observed that the adsorption capacities of synthesized siderite increased with increasing concentration of HA from 0 to 2 mg/L, which was attributed to the increase of surface adsorption sites (ferrihydrite). Similarly, a longer contact time also favored the increase of adsorption capacities. In particular, the largest adsorption capacity was achieved with a contact time of 66 h (2 mg/L HA). In addition, the adsorption capacities of synthesized siderite decreased with increasing concentration of HA from 2 to 10 mg/L, which suggested that the HA may affect P adsorption by competitively occupying the sites on the mineral surface or through increasing electrostatic repulsion. In addition, the effect of the dissolved oxygen (DO) on the adsorption of phosphate by synthesized siderite was also investigated (Supplementary Fig. 6c). The results revealed that siderite without oxidation has little adsorption capacity.

To investigate the removal mechanism of phosphate in the reaction, element mapping of the products generated from the synthesized siderite after reacting with HA and the adsorbed phosphate was conducted (Fig. 7). The distribution of element P (Fig. 7e) was consistent with the element Fe (Fig. 7b), which indicated that the new minerals (ferrihydrite and goethite) were the major mineralogical component for phosphate adsorption. Ferrihydrite and goethite provide a number of adsorption sites for ions on their surface, which allows both adsorption and co-precipitation of ions, and this is consistent with previous reports that phosphate forms predominantly a surface co-precipitate on hematite^56^. Hence, the influence of HA (0–2 mg/L) on promoting phosphate adsorption was indirectly by the creation of new mineral phases. However, a high concentration of HA also shows competitive adsorption with phosphate.

The adsorption capacity of siderite depends upon its oxidation state, which is strongly influenced by the exposure to, or presence of, oxidants (e.g. H_2_O_2_). Hence, the mechanism of phosphate adsorption on siderite with H_2_O_2_ should be different from HA. In this study, the effect of H_2_O_2_ on the adsorption of phosphate on siderite is shown in Supplementary Fig. 6b–e. It was observed that the adsorption capacities of siderite increased with increasing concentration of H_2_O_2_ from 0 to 0.2 mmol/L, which was attributed to the increase of surface adsorption sites, arising from the increase of Fe^3+^ (2FeCO_3_ + H_2_O_2_ + 6 H^+^ = 2Fe^3+^+2CO_2_ + 4H_2_O). However, the adsorption capacities of siderite decreased as the concentration of H_2_O_2_ increased from 0.2 to 1 mmol/L, which was attributed to the fact that the slow oxidation could increase Fe^2+^ activity (in-situ Fe^3+^) to the greatest extent. In the case of contact time, a longer contact time also favored adsorption, where the largest adsorption capacity was achieved with a contact time of 30 h (0.2 mmol/L).

In order to determine the phosphate species of the surface complex on the precipitates (Supplementary Fig. 7a–f), the phosphate-adsorbed products of siderite after reacting with HA (Supplementary Fig. 7a, b) and H_2_O_2_ (Supplementary Fig. 7a–c) were carefully studied by FTIR and XPS. The results revealed that a bidentate binuclear complex is the main phosphate species on the precipitate surface of siderite-HA and siderite-H_2_O_2_. The difference between them is that the phosphate species of siderite-HA is dominated by a diprotonated complex, while in contrast, a monoprotonated complex is the main phosphate species on the precipitate surface of siderite-H_2_O_2_. The detailed FTIR analysis of these are given in the Supplementary Fig. 7.

### Environmental significance

In the process of Fe-mineral tranformation, atomic Fe needs sufficient energy in order to overcome chemical barriers, usually requiring environmentally demanding conditions, such as high temperature, pressure, specific pH, and high Fe concentrations. In this study, it has been shown that humic acid can chelate ferrous ions to form a Fe(II)-HA complex, which keeps Fe atoms within an effective distance between each other. This enables the formation of mesocrystals and new mineral phases by attachment. These findings are important in understanding the pathway of Fe-mineral transformation in the natural environment. The results not only support the supposition that the mesocrystal is a necessary precursor to the single-crystal attachment, but also confirm a fundamental step for crystal growth.

The Fe-mineral transformation has special significance for the cycle of phosphate. Due to the HA-induced transformation of siderite to ferrihydrite and goethite, the influence of HA on phosphate is through both competitive adsorption and the indirect promoting of adsorption. We believe that these results are beneficial to a greater understanding of the impact of humic acid on the biogeochemical cycling of phosphate, iron-bearing mineral transformation, as well as environmental remediation.

## Methods

### Materials

The siderite was prepared by mixing solutions of ferrous sulfate (FeSO_4_·7H_2_O) and anhydrous sodium bicarbonate (Na_2_CO_3_), and autoclaving at 130 °C, according to methods that have been previously published^57^. For our experiments, firstly, two solutions were prepared: solution A: anhydrous Na_2_CO_3_ (3.816 g) was dissolved in 80 mL of DI water; solution B: ascorbic acid (6.336 g) was dissolved in 80 mL of DI water, followed by the addition of FeSO_4_·7H_2_O (3.336 g). Nitrogen was pumped continuously into the solutions A and B. Then, the two solutions were mixed with continuous stirring for 15 mins, and the final Fe^2+^ concentration in the mixed solution was 0.075 mol/L. The mixed solution was transferred into a Teflon-lined stainless-steel autoclave, which was maintained at 130 °C for 4 h. Finally, the precipitate was freeze-dried for 12 h. All chemical reagents used in the experiment were of analytical grade.

### Jar tests

The effects of humic acid (HA: CAS: 68131-04-4; 0–10 mg/L) in the presence (aerobic) and relative absence (anaerobic) of oxygen, and hydrogen peroxide (H_2_O_2_: CAS: 7722-84-1; 0–1 mmol/L) on the mineral transformation were investigated. The oxygen content of aerobic (4 mg/L) and anaerobic (1 mg/L) HA simulated water was measured by a dissolved oxygen electrode. The concentration of total Fe and Fe^2+^ were measured by Inductively Coupled Plasma (ICP) and spectrophotometer at 510 nm (UV-2600, Shimadzu, Japan), respectively.

Batch experiments were carried out using a constant dose of 0.5 g/L (siderite/solution) by adding synthesized siderite (0.025 g) into 50 mL solution. The reaction bottles were placed on a magnetic stirrer with a temperature controller at a stirring rate of 300 rpm. After aging for a set time (1 day and 1 week), the precipitate was freeze-dried for 12 h and collected.

The influence of products generated from mineral transformation on phosphate adsorption was investigated. The desired phosphate concentration (50 mg P/L) was obtained with a pH value of 7, which was prepared by adding 0.2197 g KH_2_PO_4_ into 1 L deionized water (the pH value was buffered to the neutral condition with 0.1 mol/L NaOH). The phosphate concentration after adsorption in the solution was determined by the molybdenum blue method with a spectrophotometer at 700 nm (UV-2600, Shimadzu, Japan).

### Sample analysis and data evaluation

An X-ray diffractometer (XRD, X’Pert PRO MPD, PANalytical, Netherlands) was utilized to analyze the phase composition of the siderite sample with 40 kV and 40 mA at a scan speed of 4°/min. Phase identification was carried out by comparison with reference spectra of the International Centre for Diffraction Data (ICDD).

A field emission scanning electron microscope (SEM, SU-8020, HITACHI, Japan) was used to observe and analyze the morphology of the samples, and all samples were sprayed with gold for 30 s. Field emission transmission electron microscopy (TEM, JEM-2100F, JEOL, Japan) was used to analyze the crystallinity (lattice fringes). All samples were placed in the ultrasonic instrument for 10 min, making sure that the nanoparticles were well dispersed on the Cu grid. The distribution of elements and crystallinity were investigated by element mapping and selected area electron diffraction (SAED), respectively. Each sample was mixed with alcohol and deposited on a Cu grid.

X-ray photoelectron spectroscopy (XPS, PHI Quantera II, ULVAC, Japan) measurements were conducted with a Thermo Escalab 250 electron spectrometer using 150 W Al-Kα radiations. Fourier transformed infrared spectra (FTIR, NICOLET-8700, Thermo Fisher Scientific, America) was used to analyze the surface functional groups of the siderite sample. The measurement resolution was set at 4 cm^−1^ and the spectra were collected in the range of 600–4000 cm^−1^.

Sample analysis by three-dimensional fluorescence excitation-emission matrix spectrometry (3D-EEM, FluoroMax Series, HORIBA, Japan) was conducted to provide further semi-quantitative information concerning the HA in water samples.

## Supplementary information

Supplementary Information

## Data Availability

All data analyzed during this study are included in this published article (and its Supplementary Information).

## References

[1] Chassé AW, Ohno T (2016). Higher molecular mass organic matter molecules compete with orthophosphate for adsorption to iron (oxy)hydroxide. Environ. Sci. Technol..

[2] Yang Y, Wang S, Xu Y, Zheng B, Liu J (2016). Molecular-scale study of aspartate adsorption on goethite and competition with phosphate. Environ. Sci. Technol..

[3] Frey PA, Reed GH (2012). The ubiquity of iron. ACS Chem. Biol..

[4] Tagliabue A (2017). The integral role of iron in ocean biogeochemistry. Nature.

[5] Zou Y (2016). Environmental remediation and application of nanoscale zero-valent iron and its composites for the removal of heavy metal ions: a review. Environ. Sci. Technol..

[6] Ludwig JR, Zimmerman PM, Gianino JB, Schindler CS (2016). Iron(III)-catalysed carbonyl-olefin metathesis. Nature.

[7] Okamura M (2016). A pentanuclear iron catalyst designed for water oxidation. Nature.

[8] Navrotsky A (2004). Energetic clues to pathways to biomineralization: precursors, clusters, and nanoparticles. Proc. Natl Acad. Sci. USA.

[9] Navrotsky A (2011). Nanoscale effects on thermodynamics and phase equilibria in oxide systems. Chemphyschem.

[10] Ostwald W (1897). Studien über die bildung und umwandlung fester körper. Z. Phys. Chem..

[11] Zhang X (2017). Direction-specific interaction forces underlying zinc oxide crystal growth by oriented attachment. Nat. Commun..

[12] Zhu C (2018). In-situ liquid cell transmission electron microscopy investigation on oriented attachment of gold nanoparticles. Nat. Commun..

[13] Li D (2012). Direction-specific interactions control crystal growth by oriented attachment. Science.

[14] De Yoreo JJ (2015). Crystallization by particle attachment in synthetic, biogenic, and geologic environments. Science.

[15] Kondrat SA (2016). Stable amorphous georgeite as a precursor to a high-activity catalyst. Nature.

[16] Xu YF (2018). Microscopic structure of the polymer-induced liquid precursor for calcium carbonate. Nat. Commun..

[17] Guo H, Ren Y, Liu Q, Zhao K, Li Y (2013). Enhancement of arsenic adsorption during mineral transformation from siderite to goethite: mechanism and application. Environ. Sci. Technol..

[18] Xing BB (2017). Removal of phosphate from aqueous solution by activated siderite ore: preparation, performance and mechanism. J. Taiwan Inst. Chem. Eng..

[19] Picard A, Kappler A, Schmid G, Quaroni L, Obst M (2015). Experimental diagenesis of organo-mineral structures formed by microaerophilic Fe(II)-oxidizing bacteria. Nat. Commun..

[20] Monteiro MJ (2018). Order from disorder through dissipation of free energy. Nat. Nano.

[21] Silva AKD (2018). Phase nucleation through confined spinodal fluctuations at crystal defects evidenced in Fe-Mn alloys. Nat. Commun..

[22] Zierenberg J, Schierz P, Janke W (2017). Canonical free-energy barrier of particle and polymer cluster formation. Nat. Commun..

[23] Wallace AF (2013). Microscopic evidence for liquid-liquid separation in supersaturated CaCO_3_ solutions. Science.

[24] Habraken WJEM (2013). Ion-association complexes unite classical and non-classical theories for the biomimetic nucleation of calcium phosphate. Nat. Commun..

[25] Matamoros-Veloza A (2018). A highly reactive precursor in the iron sulfide system. Nat. Commun..

[26] Chen KY (2016). Stabilization of natural organic matter by short-range-order Fe hydroxides. Environ. Sci. Technol..

[27] Nudelman F (2010). The role of collagen in bone apatite formation in the presence of hydroxyapatite nucleation inhibitors. Nat. Mater..

[28] Song RQ, Colfen H (2010). Mesocrystals-ordered nanoparticle superstructures. Adv. Mater..

[29] Kato T (2010). Polymer/calcium carbonate layered thin-film composites. Adv. Mater..

[30] Gebauer D, Cölfen H, Verch A, Antonietti M (2010). The multiple roles of additives in CaCO_3_ crystallization: a quantitative case study. Adv. Mater..

[31] Cantaert B (2012). Think positive: phase separation enables a positively charged additive to induce dramatic changes in calcium carbonate morphology. Adv. Funct. Mater..

[32] Lenders JJM (2014). A bioinspired coprecipitation method for the controlled synthesis of magnetite nanoparticles. Cryst. Growth Des..

[33] Suzuki M (2009). An acidic matrix protein, pif, is a key macromolecule for nacre formation. Science.

[34] Fu G, Valiyaveettil S, Wopenka B, Morse DE (2005). CaCO_3_ biomineralization: acidic 8-kDa proteins isolated from aragonitic abalone shell nacre can specifically modify calcite crystal morphology. Biomacromolecules.

[35] Metzler RA (2010). Nacre protein fragment templates lamellar aragonite growth. J. Am. Chem. Soc..

[36] Weiss IM, Kaufmann S, Mann K, Fritz M (2000). Purification and characterization of perlucin and perlustrin, two new proteins from the shell of the mollusc haliotis laevigata. Biochem. Biophys. Res. Commun..

[37] Sutter E (2016). In situ microscopy of the self-assembly of branched nanocrystals in solution. Nat. Commun..

[38] Funnell, N. P. et al. Nanocomposite structure of two-line ferrihydrite powder from total scattering. *Commun. Chem*. **3**, 22 (2020). 10.1038/s42004-020-0269-2 PMC9814407 36703415

[39] Wang LJ, Putnis CV, Ruiz Agudo E, Hovelmann J, Putnis A (2015). In situ imaging of interfacial precipitation of phosphate on goethite. Environ. Sci. Technol..

[40] Long M (2016). Phosphate changes effect of humic acids on TiO_2_ photocatalysis: from inhibition to mitigation of electron-hole recombination. Environ. Sci. Technol..

[41] Banfield JF, Welch SA, Zhang H, Ebert TT, Penn RL (2000). Aggregation-based crystal growth and microstructure development in natural iron oxyhydroxide biomineralization products. Science.

[42] Qu XF, Yao QZ, Zhou GT, Fu SQ, Huang JL (2010). Formation of hollow magnetite microspheres and their evolution into durian-like architectures. J. Phys. Chem. C.

[43] Yu SH, Colfen H, Antonietti M (2003). Polymer-controlled morphosynthesis and mineralization of metal carbonate superstructures. J. Phys. Chem. B.

[44] Lv YD, Wang H, Wang X, Bai J (2009). Synthesis, characterization and growing mechanism of monodisperse Fe_3_O_4_ microspheres. J. Cryst. Growth.

[45] Zhang J, Huang F, Lin Z (2010). Progress of nanocrystalline growth kinetics based on oriented attachment. Nanoscale.

[46] Tang ZY, Kotov NA, Giersig M (2002). Spontaneous organization of single CdTe nanoparticles into luminescent nanowires. Science.

[47] Sampanthar JT, Zeng HC (2002). Arresting butterfly-like intermediate nanocrystals of β-Co(OH)_2_ via ethylenediamine-mediated synthesis. J. Am. Chem. Soc..

[48] Lupulescu AI, Rimer JD (2014). In situ imaging of silicalite-1 surface growth reveals the mechanism of crystallization. Science.

[49] Nielsen MH, Aloni S, De Yoreo JJ (2014). In situ TEM imaging of CaCO_3_ nucleation reveals coexistence of direct and indirect pathways. Science.

[50] Chen Q (2015). Interaction potentials of anisotropic nanocrystals from the trajectory sampling of particle motion usingin situLiquid phase transmission electron microscopy. ACS Cent. Sci..

[51] Yuwono VM, Burrows ND, Soltis JA, Lee Penn R (2010). Oriented aggregation: formation and transformation of mesocrystal intermediates revealed. J. Am. Chem. Soc..

[52] Tan BJ, Klabunde KJ, Sherwood PMA (2002). X-ray photoelectron spectroscopy studies of solvated metal atom dispersed catalysts. Monometallic iron and bimetallic iron-cobalt particles on alumina. Chem. Mater..

[53] Petsev DN, Chen K, Gliko O, Vekilov PG (2003). Diffusion-limited kinetics of the solution-solid phase transition of molecular substances. Proc. Natl Acad. Sci. USA.

[54] Giuffre AJ, Hamm LM, Han N, De Yoreo JJ, Dove PM (2013). Polysaccharide chemistry regulates kinetics of calcite nucleation through competition of interfacial energies. Proc. Natl Acad. Sci. USA.

[55] Shu Z (2019). Solar irradiation induced transformation of ferrihydrite in the presence of aqueous Fe2+. Environ. Sci. Technol..

[56] Elzinga EJ, Huang JH, Chorover J, Kretzschmar R (2012). ATR-FTIR spectroscopy study of the influence of pH and contact time on the adhesion of Shewanella putrefaciens bacterial cells to the surface of hematite. Environ. Sci. Technol..

[57] Golden DC (2000). An experimental study on kinetically‐driven precipitation of calcium‐magnesium‐iron carbonates from solution: implications for the low‐temperature formation of carbonates in martian meteorite Allan Hills 84001. Meteorit. Planet. Sci..

