# Supplementary material for: Transformation of siderite to goethite by humic acid in the natural environment
Source: Commun Chem. 2020 Mar 25;3:38. doi: 10.1038/s42004-020-0284-3 (PMC9814924; doi:10.1038/s42004-020-0284-3)
Supplement: Supplementary file 1 — Supplementary Information [file 42004_2020_284_MOESM1_ESM.pdf]

Supplementary Information

Transformation of siderite to goethite by humic acid in the natural environment

Bobo Xing[1], Nigel Graham[2], Wenzheng Yu[1,2*]

[1] Key Laboratory of Drinking Water Science and Technology, Research Center for Eco-Environmental Sciences, Chinese Academy of Sciences, Beijing, China, 10086

[2] Department of Civil and Environmental Engineering, Imperial College London, South Kensington Campus, London SW7 2AZ, UK

wzyu@rcees.ac.cn, n.graham@imperial.ac.uk

Supplementary Methods; the growth process of siderite

To understand the growth process of the siderite, we carried out a lower-temperature experiment at 100 °C for 2 h. A variety of particles (Supplementary Figure 1a) with different shapes (rods, peanuts, cucurbit, and approximate spheres) were observed. In combination with the interpretation of the aggregation mechanism[1,2], the siderite growth process starts with elongated rod crystals (Supplementary Figure 1b), followed by self-similar fanning out from the center (Supplementary Figure 1c), and then anisotropic cucurbit-shaped aggregates were formed on the fractal faces (Supplementary Figure 1d), and finally the formation of symmetrical globular aggregates (Figure 1b).

Further evidence of mineral transformation from siderite to goethite induced by humic acid

The experimental results demonstrated that the spherical substance of small size was ferrihydrite. In order to further determine the phase of the large size spherical substance, the lattice fringes and SAED spots of these large size spherical substances were observed by high-resolution TEM (Supplementary Figures 2e and 2f) and SAED (Supplementary Figure 2d). The lattice fringes of the substances (Supplementary Figure 2e) correspond to the (006) and (110) planes in siderite, with d-spacings of 2.53 Å and 2.35 Å, respectively. The SAED spots demonstrated that the spherical substance was a single crystal of siderite (Supplementary Figure 2d). Moreover, it can be clearly seen from the SAED pattern that the sphere orientation of the base is nearly perpendicular

to the [100] direction. The calculated (012), $(01\bar{4})$, and $(00\bar{6})$ lattice planes coincide with the siderite unit cell structure ([space group $R\bar{3}c$ (167)] with lattice constants a = 4.69 Å and c = 15.38 Å), confirming that the sphere orientation of the base is nearly perpendicular to the a-axis direction.

In order to reveal the growth and polymerization process of goethite, the lattice fringes of the small size spherical substance (Supplementary Figure 2f) between the siderite and the rod-shaped substances (Supplementary Figure 2f) was observed, corresponding to the (201) planes in goethite, with d-spacings of 3.42 Å. In addition, the lattice fringes of the rod-shaped substances in Supplementary Figure 1f correspond to the (301) planes in goethite, with d-spacings of 2.71 Å. The above two points implied that the small size spherical substances between siderite and goethite were spherical nanoparticles of goethite, which were formed by structural rearrangement of ferrihydrite[3].

The XPS spectra of O 1s demonstration for the mineral transformation.

The XPS spectra of O 1s by the effect of HA and H_2O_2 are shown in Supplementary Figure 3. The O 1s peaks which are assigned to $FeCO_3$ locate at 529.8 ± 0.2 eV in Supplementary Figure 3b. The O 1s spectra by the effect of HA and H_2O_2 can be seen at 530.6 eV and 530.3 eV in Supplementary Figure 3, which are wider than that of siderite (529.8 eV). In order to reveal the reaction mechanism, their detailed O 1s analyses of siderite (Supplementary Figure 3b) by the effect of HA (Supplementary Figure 3c) and H_2O_2 (Supplementary Figure 3d) were demonstrated. The O 1s peaks of

FeOO*H and FeO*OH (Supplementary Figure 3c) are located at 531.4 ± 0.2 and 529.6 ± 0.2 eV, respectively; the peak of CO*OH located at 530.8 ± 0.2 eV (Supplementary Figure 3c) is attributed to the carboxyl of HA. The peak at 531.1 ± 0.2 eV in Supplementary Figure 3d of siderite after reacting with H_2O_2 are different with that of siderite after reacting with HA, which may result from the formation of $Fe(OH)_3$. The O 1s peaks are assigned to CO*OH located at 530.5 ± 0.2 eV in Supplementary Figure 3d of siderite after reacting with H_2O_2, which may result from the oxidative product of ascorbic acid.

Structure characterization of phosphate complexes on the precipitates

FTIR spectroscopy is a useful tool to characterize phosphate complexes formed on Fe(III)-(hydr)oxide mineral surfaces, including ferrihydrite[4], goethite[5,6], and hematite[7]. In order to reveal the mechanisms of phosphate removal under different conditions, the phosphate-adsorbed products of siderite after reacting with different concentration of HA (Supplementary Figure 7a-B) and H_2O_2 (Supplementary Figure 7a-C) were carefully investigated with FTIR.

The FTIR spectra of synthesized siderite are shown in Figure 1f and Supplementary Figure 7a-A. It can be observed that the CO_3 vibration bands of siderite were located at approximately 884 cm^{-1} (out-of-plane bending vibration band), 765 cm^{-1} (in-plane bending vibration band) and 1,435 cm^{-1} (stretching vibration band), respectively. The structure of functional groups is shown in Figure 1f. The vibration band at 1,240 cm^{-1} (Supplementary Figure 7a-B) was attributed to the C-C stretching vibrations of humic acid[8]. The stretching vibration of C-C (linked with C=O bond) enhanced with the

increase of the electron cloud density of the C-C bond, which was attributed to the conjugation effect between C=O and the aromatic ring. However, it can be observed that four vibrations including the OH stretching vibration (3,618 cm^{-1}), FeO–OH stretching vibration (3,081 cm^{-1}), δ(OH), and γ(OH) deformation vibration (892 and 770 cm^{-1}) only appeared in Supplementary Figure 7a-B, which were attributed to the goethite[5]. The vibration band at 1,000 cm^{-1} (Supplementary Figure 7a-B and 7a-C) was attributed to the Fe-O-P stretching vibrations of phosphate.

The relationship between the symmetry of the phosphate complex and the number of IR active bands is useful for demonstrating the species of the complex on the surface of siderite. Comparing the results of surface complex spectra reported by Huang[8], Elzinga[7] and Tejedor-Tejedor[5], the P-$(OH)_2$ symmetrical stretching vibration was approximately located in the range of 950-990 cm^{-1}, the P-OH asymmetric stretching vibration was approximately located in the range of 900-950 cm^{-1}, and the PO_3 asymmetrical stretching vibration band and the PO_2 symmetrical stretching vibration were located at approximately 1,130 cm^{-1} and 1,100 cm^{-1}, respectively. The P-O-Fe stretching vibration was approximately located in the range of 990-1,025 cm^{-1}, which was identified as a monodentate mononuclear complex, and the Fe-O-P-O-Fe stretching vibration was approximately located in the range of 1,025-1,075 cm^{-1}, which was identified as a monodentate binuclear (bridging) fashion complex.

In order to determine the phosphate species of the surface complex on the precipitates of siderite after the reaction, a peak fitting of the IR spectra (900-1,150 cm^{-1}) was performed (Supplementary Figures 7c and 7e). The spectra of three precipitates were

well reproduced with six Gaussian v_3 peaks, which indicated that two different phosphate species were present on the precipitate surface. The six Gaussian v_3 peaks were preliminarily identified as PO_3, PO_2, Fe-O-P-O-Fe, P-O-Fe, P-$(OH)_2$, and P-OH, respectively. It is believed that at least one of the two different phosphate species was a monoprotonated complex, and another phosphate species was a diprotonated complex. There is one significantly Gaussian v_3 peak at 969 cm $^{-1}$ (Supplementary Figure 7c), which suggested that this is the P-$(OH)_2$ symmetrical stretching vibration band[7]. Furthermore, the relative strength of P-$(OH)_2$ is greater than another spectrum (Supplementary Figure 7e), which implied that the phosphate species on the precipitate surface of siderite-HA is more likely to be a diprotonated complex. Meanwhile, the Gaussian v_3 peaks at 1,056 cm $^{-1}$ (Supplementary Figure 7c) and 1,054 cm $^{-1}$ (Supplementary Figure 7e) are identified as the Fe-O-P-O-Fe vibration band. As the vibration strength of Fe-O-P-O-Fe is higher than the P-O-Fe (Supplementary Figure 7c and 7e), this suggests that the bidentate binuclear complex is the main phosphate species on the precipitate surface of siderite-HA and siderite-H_2O_2.

The surface complexes were investigated by XPS analysis. The P 2p XPS spectra with the effect of HA and H_2O_2 are shown in Supplementary Figure 7. The P 2p peaks located at 133.5 ± 0.2 and 132.2 ± 0.2 eV are assigned to Fe-O-PHO_3 and Fe-O-PH_2O_3, respectively[9]. This suggests that the siderite-HA is dominated by a diprotonated complex, while in contrast, the monoprotonated complex is the main phosphate species on the precipitate surface of siderite-H_2O_2. These conclusions are consistent with FTIR analysis.

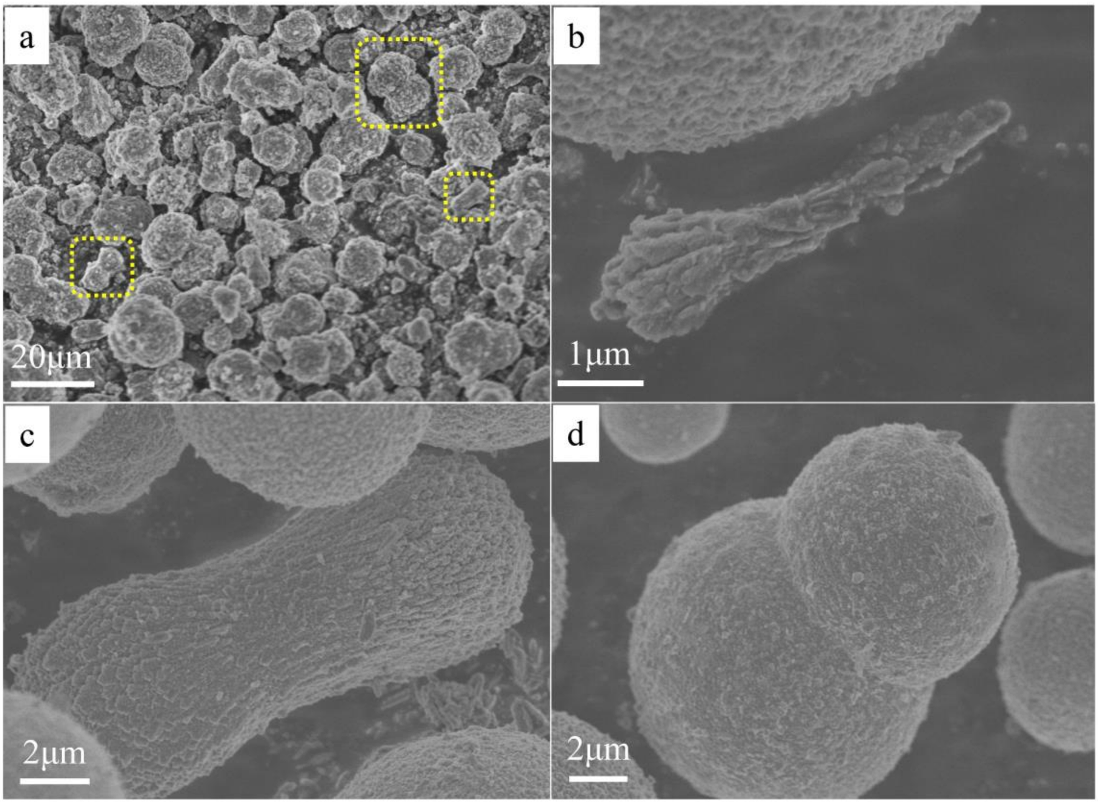

Supplementary Figure 1. Morphology of synthesized siderite. (a) Representative SEM images of the synthesized siderite obtained through hydrothermal reaction at 100 °C for 2 h, containing different shapes: rod (b), peanut (c), and cucurbit (d).

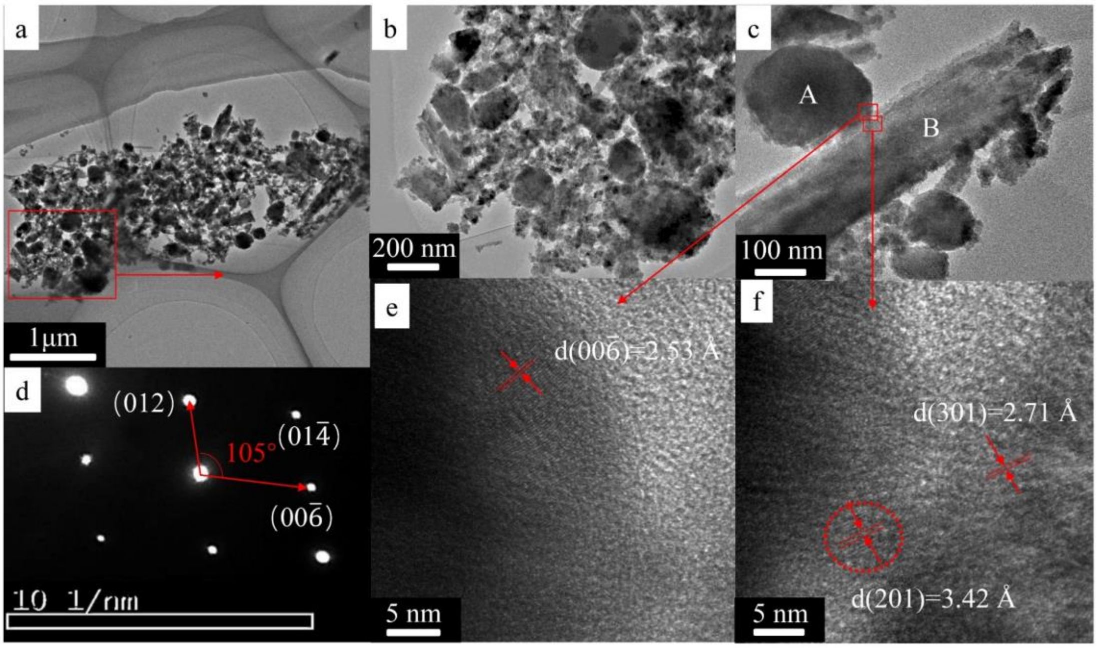

Supplementary Figure 2. Morphology and crystallinity of products. Representative TEM (a, b, and c), HRTEM (e and f), and SAED (d) images of the precipitate of siderite after reacting with HA; Samples were freeze-dried for 12 h after 7 days of aging.

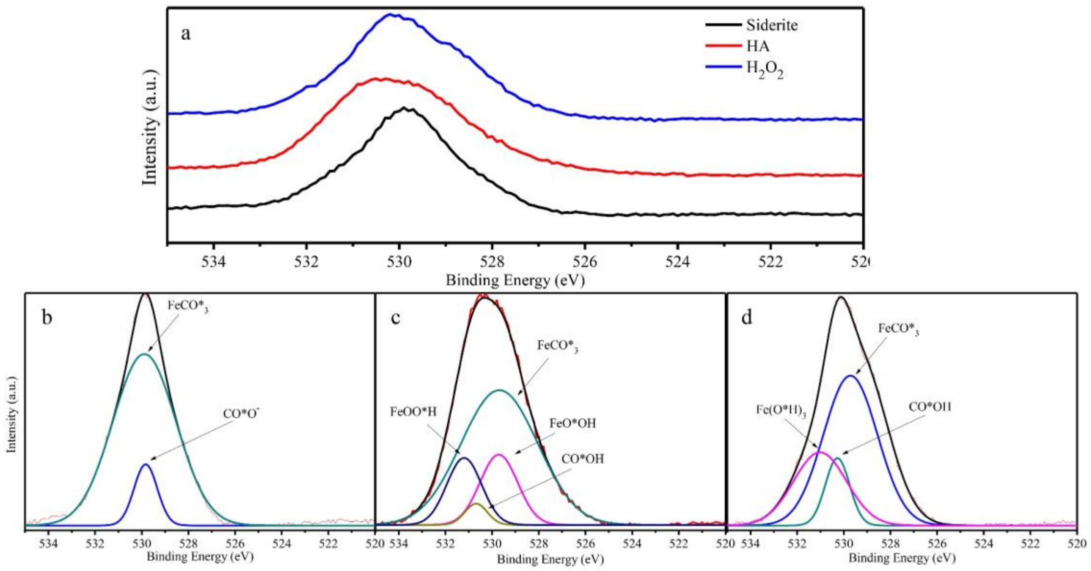

Supplementary Figure 3. The XPS spectra of O 1s. The survey and high-resolution scans of XPS spectra of siderite after reacting with HA and H_2O_2. (a) Total survey scans of O 1s; (b) O 1s peaks of siderite; (c) O 1s peaks of siderite-HA; (d) O 1s peaks of siderite-H_2O_2. Samples were taken after 7 days of aging.

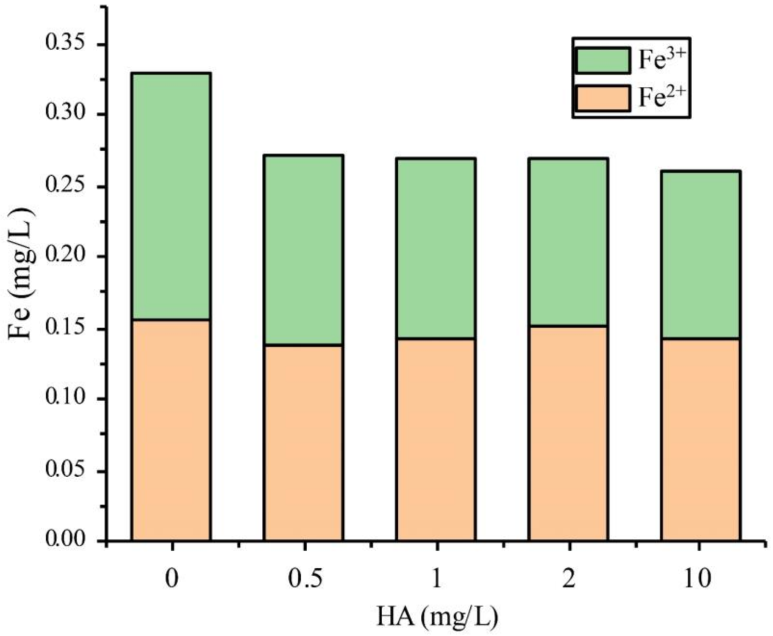

Supplementary Figure 4. The concentration of Fe in the solution. The sample of solution was taken after siderite reacting with different concentration of HA.

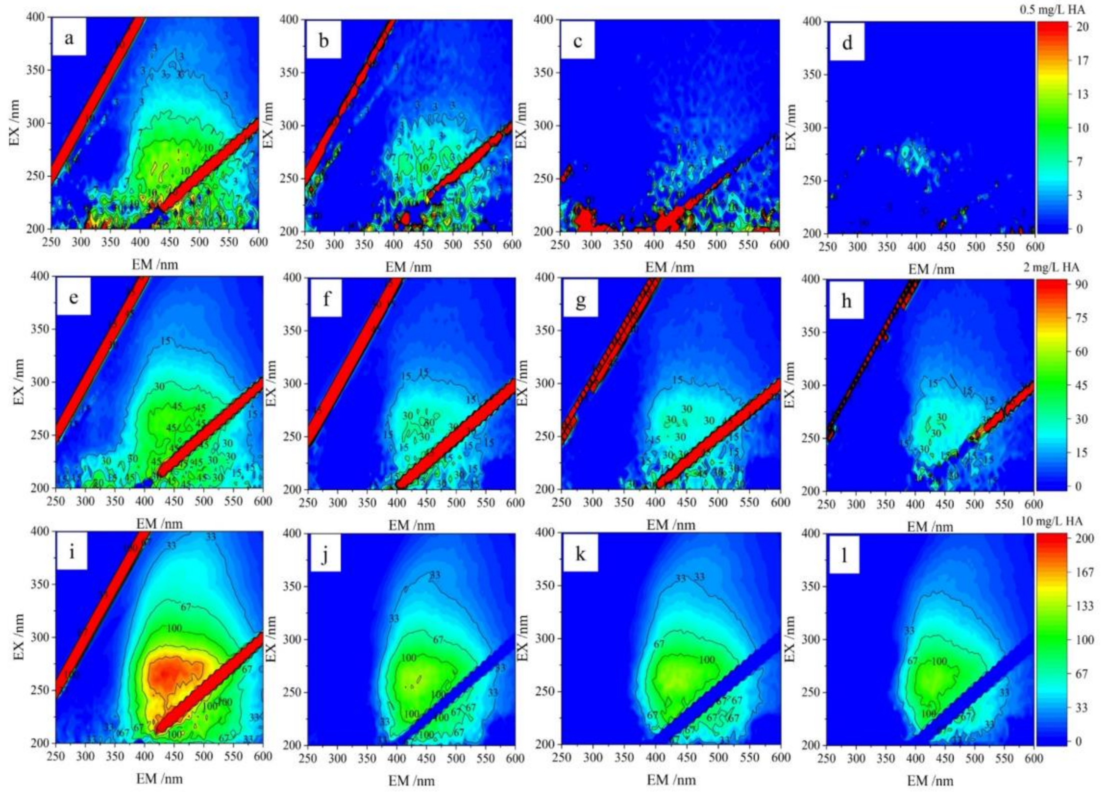

Supplementary Figure 5. Fluorescence spectra of HA. The EEM of synthesized siderite after reacting with HA at different times (b: 2h-0.5 mg/L HA, c: 6h-0.5 mg/L HA, d: 30h-0.5 mg/L HA; f: 2h-2 mg/L HA, g: 6h-2 mg/L HA, h: 30h-2 mg/L HA; j: 2h-10 mg/L HA, k: 6h-10 mg/L HA, l: 30h-10 mg/L HA). The initial concentration of HA was 0.5 (a), 2 (e), and 10 (i) mg/L. (T=298 K, pH=7).

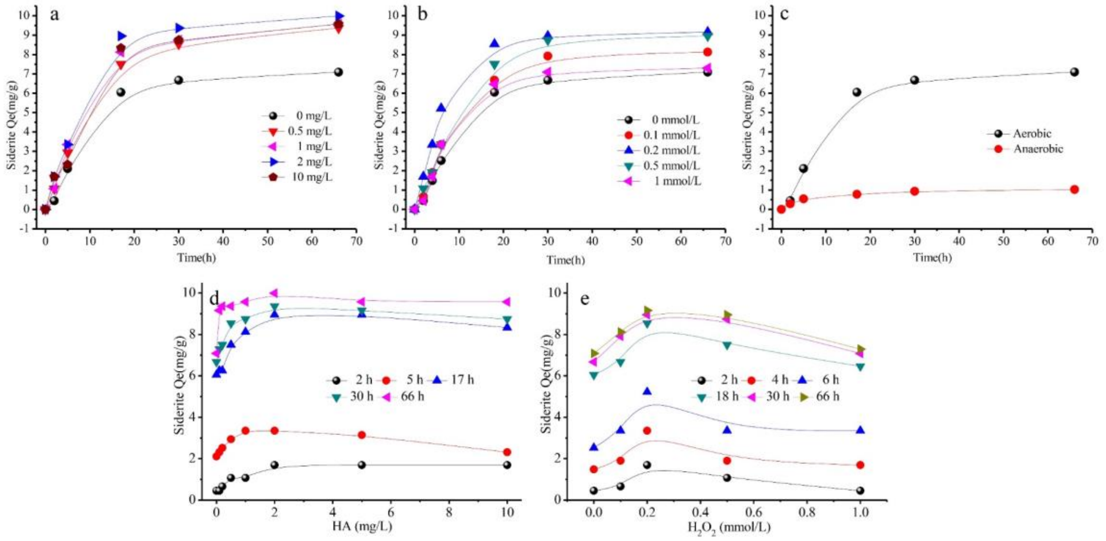

Supplementary Figure 6. The adsorption of phosphate. Effect of reaction time (a and b), dissolved oxygen (c), and concentration (d and e) of HA and H_2O_2 on the adsorption of phosphate by siderite, respectively. (T=298 K, pH=7, C_0=5 mg/L)

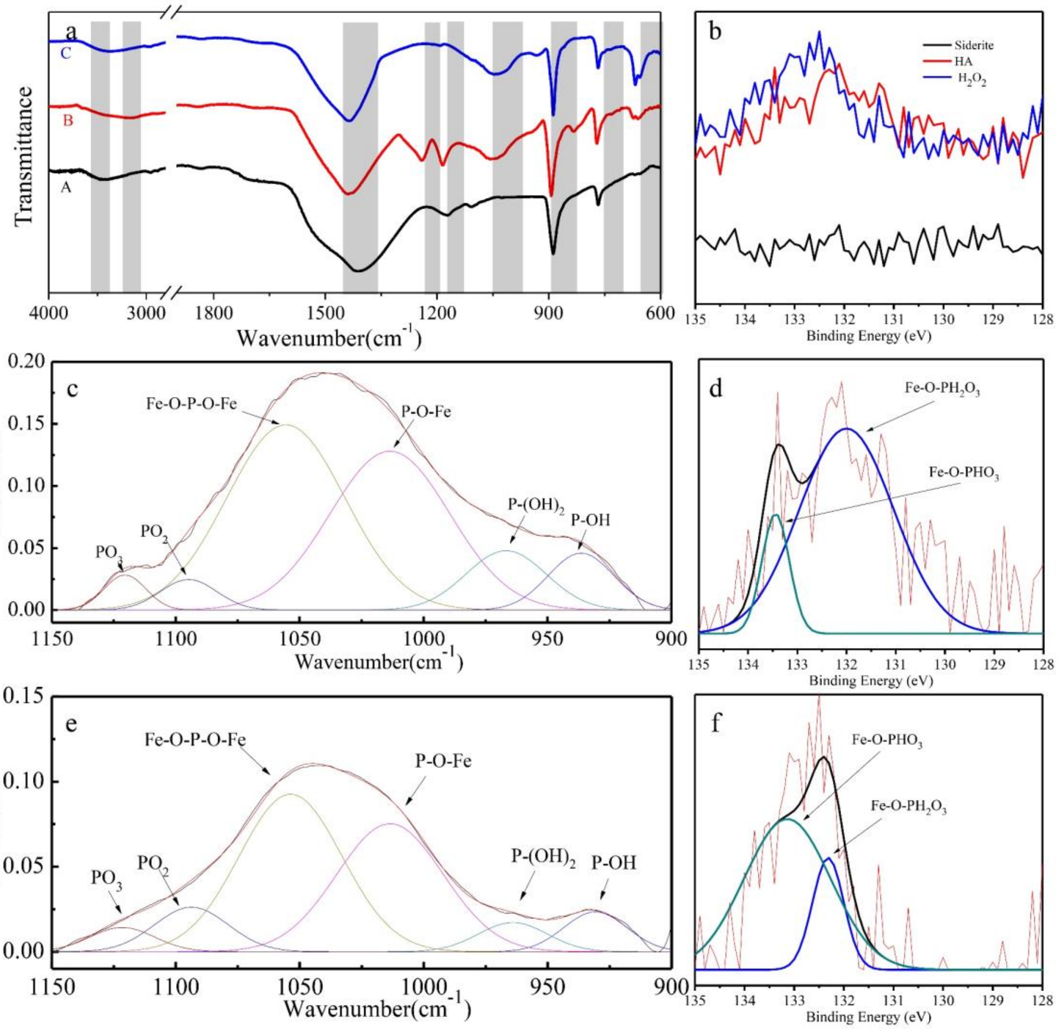

Supplementary Figure 7. Structure characterization of phosphate complexes. The FTIR spectra (a) of synthesized siderite (spectrum A) after reacting with HA-P (spectrum B), H_2O_2-P (spectrum C); (c) Curve-fitting analysis of the FTIR spectrum B; (e) Curve-fitting analysis of the FTIR spectrum C. The survey and high-resolution scans of XPS spectra of siderite by the effect of HA and H_2O_2; (b) Total survey scans of P 2p; (d) P 2p peaks of siderite-HA; (f) P 2p peaks of siderite-H_2O_2. The initial phosphate concentration was 5 mg/L, at pH 7 (298 K).

Supplementary References

1 Zhou, G. T., Yao, Q. Z., Ni, J. & Jin, G. Formation of aragonite mesocrystals and implication for biomineralization. *Am. Mineral.* **94**, 293-302 (2009).

2 Qu, X. F., Yao, Q. Z., Zhou, G. T., Fu, S. Q. & Huang, J. L. Formation of hollow magnetite microspheres and their evolution into durian-like architectures. *J. Phys. Chem. C* **114**, 8734-8740 (2010).

3 Li, D. S. *et al.* Growth mechanism of highly branched titanium dioxide nanowires via oriented attachment. *Cryst. Growth. Des.* **13**, 422-428 (2013).

4 Wang, X. M. *et al.* Effect of ferrihydrite crystallite size on phosphate adsorption reactivity. *Environ. Sci. Technol.* **47**, 10322-10331 (2013).

5 Tejedor-Tejedor, M. I. & Anderson, M. A. The protonation of phosphate on the surface of goethite as studied by CIR-FTIR and electrophoretic mobility. *Langmuir* **6**, 602-611 (1990).

6 Wang, L. J., Putnis, C. V., Ruiz Agudo, E., Hovelmann, J. & Putnis, A. In situ imaging of interfacial precipitation of phosphate on goethite. *Environ. Sci. Technol.* **49**, 4184-4192 (2015).

7 Elzinga, E. J., Huang, J. H., Chorover, J. & Kretzschmar, R. ATR-FTIR spectroscopy study of the influence of pH and contact time on the adhesion of Shewanella putrefaciens bacterial cells to the surface of hematite. *Environ. Sci. Technol.* **46**, 12848-12855 (2012).

8 Huang, J. H., Elzinga, E. J., Brechbuehl, Y., Voegelin, A. & Kretzschmar, R. Impacts of shewanella putrefaciens strain CN-32 cells and extracellular polymeric substances on the sorption of As(V) and As(III) on Fe(III)-(Hydr)oxides. *Environ. Sci. Technol.* **45**, 2804-2810 (2011).

9 Tan, B. J., Klabunde, K. J. & Sherwood, P. M. A. X-ray photoelectron spectroscopy studies of solvated metal atom dispersed catalysts. Monometallic iron and bimetallic iron-cobalt particles on alumina. *Chem.Mater.* **2**, 186-191 (2002).
